# Patterns of Sexual Dysfunction in Depression: A Population-Based Study in Sweden

**DOI:** 10.1192/j.eurpsy.2025.473

**Published:** 2025-08-26

**Authors:** J. Isung, P. Karlsson, M. Schuier, V. Johansson, K. Gembert, J. Reutfors

**Affiliations:** 1 Karolinska Institutet, Stockholm, Sweden; 2 J&J Innovative Medicine, Raritan, United States; 3 Umeå University, Umeå, Sweden

## Abstract

**Introduction:**

Previous research suggests that sexual dysfunction (SD) can both contribute to and result from depression. Additionally, evidence indicates that antidepressants may cause SD as a side-effect. However, knowledge of SD patterns in depressed individuals at the population level remains limited.

**Objectives:**

To describe and compare the prevalence and incidence of SD during a three-year period before and after a diagnosis of depression.

**Methods:**

Nationwide health registers in Sweden were used to identify patients diagnosed with a new-onset depressive episode (ICD-10: F32 and F33) in specialized healthcare between 2006 and 2014. SD was defined as having an SD diagnosis (ICD-10: F52.0-52.3) or a filled prescription of a drug aimed against SD (phosphodiesterase 5 inhibitor) for women and men separately. First, the prevalence of SD was calculated for the three-year period before and after the depression diagnosis. Second, annual incidence rates of SD were calculated by only including the first-ever SD event for each year during the same periods. Finally, in men, the annual incidence rates of SD were stratified by age groups (18–29, 30–49, and 50–65 years).

**Results:**

We identified 110,725 women (mean age 38 years) and 73,566 men (mean age 39 years) with newly diagnosed depression. Among the women, 117 had SD in the three years before the depression diagnosis, corresponding to a three-year prevalence of 0.12% (95% CI 0.10%–0.14%), whereas 192 had SD in the 3 years after the depression diagnosis, corresponding to a 3-year prevalence of 0.19%, 95% CI 0.17%–0.22%). The annual incidence of SD ranged from 0.03%–0.09% with the highest incidence in the first year after depression. Among the men, 4,299 had SD in the 3 years before the depression diagnosis, corresponding to a 3-year prevalence of 6.4% (95% CI 6.2%–6.6%), whereas 5,716 had SD in the 3 years after the depression diagnosis, corresponding to a 3-year prevalence of 8.6% (95% CI 8.4%–8.8%). The annual incidence of SD ranged from 1.4%-2.2%, with the highest incidence in the first year after depression. When stratified by age, the annual incidence of SD in men was lowest in the youngest group (18-29 years: 0.2%–0.8%) compared to the older age groups (30-49 years: 1.4%–2.6%; 50-65 years: 2.2%–3.3%).

**Conclusions:**

In this study of patients with specialist treated depression, SD was significantly more commonly diagnosed and/or treated in the three-year period after the depression diagnosis than before in both women and men. Furthermore, the incidence of SD was highest in the first year after the depression diagnosis. As expected, SD was more common among men, where it also increased with age.

**Disclosure of Interest:**

J. Isung Grant / Research support from: Affiliated with/employed at the center for Pharmacoepidemiology, Karolinska Institutet, which receives grants from several entities (pharmaceutical companies, regulatory authorities, contract research organizations) for the performance of drug safety and drug utilization studies., P. Karlsson Grant / Research support from: Affiliated with/employed at the center for Pharmacoepidemiology, Karolinska Institutet, which receives grants from several entities (pharmaceutical companies, regulatory authorities, contract research organizations) for the performance of drug safety and drug utilization studies., M. Schuier Employee of: Employee of J&J Innovation. The work on this study was part of the employment., V. Johansson Grant / Research support from: Affiliated with/employed at the center for Pharmacoepidemiology, Karolinska Institutet, which receives grants from several entities (pharmaceutical companies, regulatory authorities, contract research organizations) for the performance of drug safety and drug utilization studies., K. Gembert Grant / Research support from: Affiliated with/employed at the center for Pharmacoepidemiology, Karolinska Institutet, which receives grants from several entities (pharmaceutical companies, regulatory authorities, contract research organizations) for the performance of drug safety and drug utilization studies., J. Reutfors Grant / Research support from: Affiliated with/employed at the center for Pharmacoepidemiology, Karolinska Institutet, which receives grants from several entities (pharmaceutical companies, regulatory authorities, contract research organizations) for the performance of drug safety and drug utilization studies.

